# DynaStI: A Dynamic Retention Time Database for Steroidomics

**DOI:** 10.3390/metabo9050085

**Published:** 2019-04-30

**Authors:** Santiago Codesido, Giuseppe Marco Randazzo, Fabio Lehmann, Víctor González-Ruiz, Arnaud García, Ioannis Xenarios, Robin Liechti, Alan Bridge, Julien Boccard, Serge Rudaz

**Affiliations:** 1 School of Pharmaceutical Sciences, University of Geneva, University of Lausanne, 1206 Geneva, Switzerland; marco.randazzo@supsi.ch (G.M.R.); Victor.Gonzalez@unige.ch (V.G.-R.); Arnaud.Garcia@unige.ch (A.G.); Julien.Boccard@unige.ch (J.B.); Serge.Rudaz@unige.ch (S.R.); 2 Dalle Molle Institute for Artificial Intelligence, 6928 Manno, Switzerland; 3 Swiss Institute of Bioinformatics, 1015 Lausanne, Switzerland; fabio.lehmann87@gmail.com (F.L.); ioannis.xenarios@unil.ch (I.X.); robin.liechti@sib.swiss (R.L.); Alan.Bridge@sib.swiss (A.B.); 4 Swiss Center for Applied Human Toxicology, 4055 Basel, Switzerland; 5 Center for Integrative Genomics, University of Lausanne, 1015 Lausanne, Switzerland

**Keywords:** metabolomics, steroidomics, database, prediction

## Abstract

Steroidomics studies face the challenge of separating analytical compounds with very similar structures (i.e., isomers). Liquid chromatography (LC) is commonly used to this end, but the shared core structure of this family of compounds compromises effective separations among the numerous chemical analytes with comparable physico-chemical properties. Careful tuning of the mobile phase gradient and an appropriate choice of the stationary phase can be used to overcome this problem, in turn modifying the retention times in different ways for each compound. In the usual workflow, this approach is suboptimal for the annotation of features based on retention times since it requires characterizing a library of known compounds for every fine-tuned configuration. We introduce a software solution, DynaStI, that is capable of annotating liquid chromatography-mass spectrometry (LC–MS) features by dynamically generating the retention times from a database containing intrinsic properties of a library of metabolites. DynaStI uses the well-established linear solvent strength (LSS) model for reversed-phase LC. Given a list of LC–MS features and some characteristics of the LC setup, this software computes the corresponding retention times for the internal database and then annotates the features using the exact masses with predicted retention times at the working conditions. DynaStI is able to automatically calibrate its predictions to compensate for deviations in the input parameters. The database also includes identification and structural information for each annotation, such as IUPAC name, CAS number, SMILES string, metabolic pathways, and links to external metabolomic or lipidomic databases.

## 1. Introduction

Recent times have seen an overhaul of our understanding of fundamental biochemistry owing to ever-improving analytical techniques. The ability to perform high-resolution, multidimensional separations has allowed the field to move from compound-specific measurements to wide untargeted analyses that can in turn be fed to powerful statistical tools [1]. These advances have been particularly useful for metabolomics, where the space of possible compounds ranges widely with respect to mass, size, and chemical properties. One of the go-to implementations is liquid chromatography–mass spectrometry (LC–MS). Compounds can be separated in a first dimension by LC, relying on the chemical interactions between the compounds and the column used in the separation. Afterwards, high-resolution MS is able to resolve masses up to the nuclear mass defect, unequivocally determining which atoms are present in a compound.

Steroidomics is especially affected by this [2]. The approach targets a family of compounds appearing in diverse metabolic pathways and with different biological roles, yet possessing a high similarity in structural and chemical terms. These issues are problematic both from the MS perspective (multiple steroids share the same molecular formula rearranged with various isomeric configurations) and from the LC perspective (most chromatographic supports are unable to exhibit selectivity towards all possible spatial changes in the molecules). One could be tempted to consider MS/MS fragmentation at this point, since it provides a direct fingerprint of the structure of the compound. Unfortunately, fragmentation patterns for steroids are quite similar since the shared core structure usually leads to common unspecific fragments. In addition, in real biological samples, the quantity of certain steroids may already fall below the threshold for detection in standard MS, let alone provide an adequate number for fragmentation to observe low-quality MS/MS spectra. Hence, optimization of the chromatographic separation becomes critical and remains the main way to distinguish different candidates of biological interest. Thankfully, the retention time of different compounds is sensitive to multiple parameters and, of interest to us, the mobile phase composition gradient. By adequately tuning a gradient involving two solvents with different elution strengths, separation can be achieved for similar compounds.

Even if separation is successful enough that the LC–MS system returns a dataset of distinct experimental features, the question of identity remains. The usual procedure is to refer to a set of standards. Features in real samples measured under identical conditions as the standards can then be characterized by matching physico-chemical properties, such as their accurate mass, isotopic pattern, MS/MS spectra, and observed retention time. It is important to stress that this is not an identification, but an annotation workflow, in that one only assigns a putative identity (or quite often, a set of identities) to a certain detected feature. This distinction is because mass measurements can only determine chemical composition, and retention time can be shared within the experimental tolerance by multiple compounds, potentially not included in the reference library. A hierarchy of annotation levels is commonly used (see for instance [3] and [4]), including the following:

- Level 4: unknown.
      Exact mass.
- Level 3: chemical class.
      Exact mass, some additional physico-chemical properties.
- Level 2: annotated metabolite.
      Two orthogonal properties, from external database/in-silico calculations.
- Level 1: identified metabolites.
      Two orthogonal properties, reference standard, same laboratory.

In this context, it will be relevant to distinguish between the standard level 2 and a newly introduced level 2+. This is related to the dynamic retention time generation that is the keystone of DynaStI. The underlying model is extremely robust when using experimental parameters measured from standards, as will be shown in detail later in the article.

Jumping straight to level 1 can be challenging for an untargeted analysis in steroidomics. However, if the separation has been optimized, level 2 putative annotations are few per observed feature. A better approach is to study which features are statistically relevant by using some sort of multivariate analysis. With the level 2 annotations for those features in hand, one has a shortlist of candidates that can be subsequently confirmed or quantified in a targeted study.

The key clause is “if the separation has been optimized”. For every different gradient, optimized for every situation, one would naïvely need to characterize the whole library of standard compounds to retrieve the association between retention times and compound identities.

Thankfully, reverse-phase liquid chromatography (RPLC) on identical analytical columns and temperatures (i.e., stationary phase chemistry) is amenable to a certain amount of modeling, at least as far as the eluent in the separation is concerned. The linear solvent strength (LSS) model [5] is notably able to reproduce the retention caused by an arbitrary mixture of two given components in the mobile phase by using only two experimental parameters per compound (compare with the n measurements needed for n different gradients in a naïve approach). To estimate these LSS parameters, only two retention times for each compound in the library of standards must be measured, each under two different gradient conditions. With the LSS model in hand, a database of retention times for any other gradient configuration can be dynamically generated at any time [6,7].

The aim of DynaStI is to implement such functionality and to wrap it in a user-friendly web interface. The basic workflow takes a list of observed features, a gradient configuration, annotation tolerances, and possibly a set of calibration compounds with known retention times. The program returns the list of annotations, together with relevant chemical information about them and references to external databases.

This article will briefly review the theoretical foundations of the prediction model. It will then describe in detail the database on which DynaStI runs, including the experimental conditions and the library of standards. The concrete web implementation is given afterwards. Finally, a real application to steroidomics will be presented to illustrate the power of the software.

## 2. Theoretical overview

### 2.1. LSS Model

The key ingredient for dynamic retention time generation is having a highly accurate model for migration with a few reliable parameters. In the case of RPLC, the LSS model provides such a framework. For a mixture of two solvents, *Weak* and *Strong*, with volume fraction $\phi ={\mathrm{vol}}_{Strong}/{\mathrm{vol}}_{Weak+Strong}$, it states the empirical formula

(1) $$k=k_{w}e^{-S\phi },$$

where k is the retention factor of an analyte, so that for a mobile phase velocity $u_{0}$ the migration velocity u of a certain analyte will be

(2) $$u=\frac{u_{0}}{1+k}\;.$$

The parameters controlling the formula are $k_{w}$, the retention in pure *Weak* solvent, normally water, and S, which measures the response to increasing fraction of *Strong* solvent, generally of organic nature such as acetonitrile or methanol. In gradient chromatography, the solvent proportion ϕ changes as time t passes, and also along the position z in the column as the mobile phase moves forward,

(3) $$\phi =\phi_{0}+\Delta \phi \;\frac{t-z/u_{0}}{t_{G}}\;,$$

where $t_{G}$ is the time over which one increases the solvent gradient from $\phi_{0}$ (the starting solvent fraction at the inlet) to $\phi_{0}+\Delta \phi$.

In a column of length L, a completely unretained compound will take the so-called dead (or hold-up) time $t_{0}=L/u_{0}$ to traverse it. By integrating the position z of the analyte as a function of time, $\partial_{t}z=1/u$, one obtains the retention time for the analyte,

(4) $$t_{R}=t_{0}\left[1+\frac{1}{b}\ln\left(1+{\mathrm{bk}}_{0}\right)\right]$$

where $k_{0}=e^{-S\phi_{0}}$ is the initial retention, and

(5) $$b=\Delta \phi \;S\;\frac{t_{0}}{t_{G}}$$

is the intrinsic gradient steepness. In real scenarios, the gradient does not start immediately at the inlet of the column at z=0, but it takes a certain dwell time $t_{D}$ from the solvent mixer to the head of the column. In that case, the retention time formula should be corrected to read

(6) $$t_{R}=t_{0}\left[1+\frac{t_{D}}{t_{0}}+\frac{1}{b}\ln\left(1+b\left(k_{0}-t_{D}/t_{0}\right)\right)\right]\;.$$

In summary, by knowing the properties of the compound in a certain separation ($k_{w},S$) and the instrumental parameters ($t_{0},t_{D},t_{G},\phi_{0},\Delta \phi$), one can predict where in the chromatogram the corresponding analyte should appear.

### 2.2. Experimental Properties, QSRR, and Level 2+ Annotation

While the instrumental parameters just introduced should be readily available to the experimenter, the values of $\left(\log k_{w},S\right)$ must be determined independently for each compound in the library. These values sum up the interactions between the compound in the mobile phase and in the stationary phase. The values are thus different for each combination of mobile phase composition, stationary phase chemistry, and temperature of the analysis. With this combination fixed, one can run two different gradients, with different intrinsic steepness $\left(b_{1},b_{2}\right)$. For accurate prediction, a factor of 3 or higher is generally considered on the intrinsic steepness ratio. This approach will generate for each compound a couple of retention times $\left(t_{1},t_{2}\right)$. The LSS parameters can be recovered by numerically solving the system of equations

(7) $$t_{1}=t_{R}\left(b_{1},k_{w},S\right),\;t_{2}=t_{R}\left(b_{2},k_{w},S\right)$$

A database of $\left(\log k_{w},S\right)$ values can then be created for the given mobile and stationary phase conditions by measuring the retention times of a library of standard compounds for two different gradients.

However, this process is still limiting insofar as available standard libraries might not be as exhaustive as one would need. In that case, *in silico* computation of parameters becomes the next best approach. In particular, the so-called quantitative structure retention relationship (QSRR) [8] can predict to reasonable accuracy the LSS parameters based only on a linear model on molecular descriptors [9] of a topological/structural/quantum mechanical nature (see [10] for a review).

This does not mean that *in silico* values can replace experimental measurements. As we will see in a concrete application, the predicted values can produce retention times with errors well above the 2% threshold. This means that annotation tolerance in retention time must be relatively high for compounds where only the QSRR values are available, which in turn will produce multiple candidates for a single feature. On the other hand, when the $\left(\log k_{w},S\right)$ parameters are experimentally determined, the robustness of the LSS model gives very reliable retention time predictions. We then differentiate between level 2 annotations (candidates selected by exact mass and predicted retention time from QSRR parameters) and level 2+ annotations (candidates selected by exact mass and predicted retention time from experimental $\left(\log k_{w},S\right)$ parameters). The reason is that while not being a full level 1 identification, with the structure of the molecule confirmed by some technique such as MS/MS fragmentation, reliable level 2+ annotations typically return as few as a single candidate from the database compounds. This is not an identification (the compound could be not in the database), but should any feature of interest turn up during analysis, it will most likely reduce the final identification step to a single check.

## 3. Database

### 3.1. Steroid Library

The current DynaStI database offers dynamic retention time generation for LC separations under the following conditions:

- Stationary phase: Kinetex^TM^ (Phenomenex) C_18_ 100 Å, 2.1 × 150 × 1.7 mm,
- Weak solvent: Water + 0.1% formic acid,
- Strong solvent: Acetonitrile + 0.1% formic acid,
- Temperature: 30 °C.

Values of $\left(\log k_{w},S\right)$ have been obtained under these conditions from a library of 198 endogenous steroids, listed in the Supplementary material, of which 92 have been measured experimentally.

### 3.2. Autocalibration

The prediction capabilities of DynaStI work in the measure that the input parameters realistically represent the experimental conditions. The values of $\left(\log k_{w},S\right)$ have been carefully measured and depend only on the intrinsic properties of the compounds. However, the set of $\left(t_{0},t_{D},b\right)$ can be a source of variation if not appropriately chosen. The retention time calculated with the LSS model assumes certain ideal conditions (negligible dead volume between column and detection, perfectly linear flow, etc.) so that even if the dwell and dead times are measured as accurately as possible, it does not mean that they correspond exactly to the parameters in the LSS retention time. With this consideration in mind, an important feature of DynaStI is an autocalibration based on the retention times for a limited number of known compounds, generally present in biological samples or that can be independently analyzed as a single mixture. Suppose that we introduce a small variation in the instrumental parameters

(8) $$t_{0}\to t_{0}+{\delta t}_{0},$$

(9) $$t_{D}\to t_{D}+{\delta t}_{D},$$

(10) b→b+δb.

If the variations are small, we can approximate the corresponding transformation to the retention time with

(11) $$t_{R}\to \left(1+\frac{{\delta t}_{0}}{t_{0}}-\frac{\delta b}{b}\right)t_{R}+{\delta t}_{D}-\frac{t_{D}}{t_{0}}{\delta t}_{0}+\left(t_{0}\frac{1+b}{b}+t_{D}\right)\frac{\delta b}{b}+\frac{\delta C}{k_{0}}+O\left(\delta^{2}\right).$$

The constant δC only depends on the parameters $\left(t_{0},t_{D},b\right)$. In gradient chromatography, the initial retention $k_{0}$ is typically very large (at least on the order of $∼10^{3}$), and the term going as $1/k_{0}$ can be discarded. Since the values of S are also larger than unity, the δb terms will also be independent of S. This means that under small changes to the instrumental parameters, the retention times transform globally as

(12) $$t_{R}\to {\alpha t}_{R}+\beta$$

where the constants a and b do not depend on the particular compound.

As argued, the provided values of $\left(t_{0},t_{D},t_{G},\phi_{0},\Delta \phi \right)$ will not in general be exact and will lead to a systematic error that can spoil the annotation. However, as previously mentioned, considering a set of features of known identity, one can use them to correct the overall LSS parameters and therefore the predicted retention times. Suppose one has observed retention times $t_{R,i}^{\exp}$ for the known compounds, and the corresponding predictions are $t_{R,i}^{\mathrm{pred}}$. Assuming that the parameters used for the prediction are slightly different from the real, effective ones, we know that the following must hold

(13) $$t_{R,i}^{\mathrm{pred}}=\alpha \;t_{R,i}^{\exp}+\beta .$$

DynaStI can take a list of features of known identity and use them to estimate a and b through a least-squares fit between the provided experimental times and its internal prediction. For the rest of the compounds of the library, the program then computes a calibrated prediction $t_{R,j}^{c.\mathrm{pred}}$ with

(14) $$t_{R,j}^{c.\mathrm{pred}}=\frac{t_{R,j}^{\mathrm{pred}}-\beta }{\alpha }.$$

### 3.3. Annotation

The annotation algorithm is rather straightforward. As the list of database compounds is not excessively long by the standards of modern computers, the algorithm requires no special optimization. In addition to the instrumental parameters $\left(b,t_{0},t_{D}\right)$, the $\left(\log k_{w},S\right)$ database and a list of measured features in the form of $\left(m/z,t_{R}\right)$ couples, one should provide a list of possible adducts to be considered in the raw data. Then, for every combination of every feature $\left(m/z_{i}^{\exp},t_{R,i}^{\exp}\right)$ and every adduct producing a mass excess $m_{j}^{\mathrm{add}}$ and having charge $q_{j}^{\mathrm{add}}$, DynaStI checks whether there is any compound in the database with properties $\left(m_{k},\log k_{w,k},S_{k}\right)$ such that

(15) $$\frac{\left|q_{j}^{\mathrm{add}}\cdot m/z_{i}^{\exp}+m_{j}^{\mathrm{add}}-m_{k}\right|}{m_{k}}<{\mathrm{tol}}_{m}{,}_{\;}$$

(16) $$\frac{\left|t_{R,i}^{\exp}-t_{R,k}^{\mathrm{pred}}\right|}{t_{R,k}^{\mathrm{pred}}}<{\mathrm{tol}}_{t_{R}}{,}_{\;}$$

where $t_{R,k}^{\mathrm{pred}}$ is computed using the LSS model for the parameters of the k-th compound. The algorithm returns a list of all possible matches—the annotations themselves—for every feature. Using the correct tolerance is critical for a good annotation. When the tolerance is lower than the experimental error, the algorithm will not find the (error-affected) feature within the database. When the tolerance is too large, too many annotations per feature will be provided. Because of the inherent difference in reliability between experimental and QSRR LSS parameters, DynaStI accepts two different levels of tolerance for predictions. A discussion about the setting of these tolerance values will be provided in the application section.

### 3.4. Implementation

The DynaStI platform itself consists of two main modules. One is the DynMetID core, already introduced in [7], in charge of the low-level tasks such as the evaluation of the LSS model. The other is the DynaStI database and user interface itself, capable of taking the feature list and gradient configuration and retrieving the annotation candidates together with the relevant information stored in the database.

DynMetID is open source, written in C++, and can be run as a standalone executable. Its command line specification is given in the Supplementary material. The average user should not be too concerned about its internal details, since the DynaStI web interface takes care of the full workflow. This core program returns the list of annotations per feature in JSON format, which is especially well suited for parsing by web applications. Notice that DynMetID does not include the database, which must be supplied as an external parameter. This also allows the end user to supply their own parameters for columns/conditions different than the ones used in this article.

The DynaStI web application itself is available at https://dynasti.vital-it.ch. It has been developed in PHP and MySQL (backend) and AngularJS (frontend). The interface offers the possibility of downloading the compounds in the database, browsing them to download all the attached metadata, and most importantly, performing annotation. The input is the same required by DynMetID but given through a user-friendly graphical interface. The list of features and the optional list of calibration compounds must be given in the same format required by DynMetID, also specified in the Supplementary material. The adducts, however, can be selected from a list of common occurrences, eliminating the need to calculate the corresponding mass differences.

## 4. Experimental Results

Preliminary work on dynamic annotation [7] has demonstrated several issues, including the need for a specific study regarding the correct tolerances required for an optimal annotation with the dynamic database.

On the one hand, the error inherent to the prediction most likely outweighs that coming from measurement, owing to the high reproducibility of the chromatographic process in RPLC. This feature is, of course, superior for predictions based on *in silico* parameters. In contrast, real applications involve extensive sets of features. The overlap of isomers with similar interactions with the column will bloat the annotation list for any given feature, which can only be countered by limiting the tolerances as much as possible.

The relative performance of *in silico* (by QSRR) relative to experimentally determined $\left(\log k_{w},S\right)$ parameters was first analyzed by comparing both predictions over a set of features obtained from known standards. The response of annotation multiplicity to tolerance was studied on both the standards and a real sample containing steroids obtained in the context of the analysis of pooled human seminal liquid samples. Such a matrix was chosen because the content of steroids of the seminal liquid has not yet been thoroughly investigated in humans [11], and because it may provide relevant information about the steroidogenesis state for the prostate, testicle or seminal vesicle. Levels of steroids in such organs may be represented in semen as a mix of secretions originating from these glands. For instance, prostate cancer is correlated with the presence of UDP-glucuronosyltransferase 2B15, resulting in variation of 5α-dihydrotestosterone in the prostate [12,13]. Another example is doping abuse, which has been proven to induce impairment of steroidogenesis in testis by suppression of the luteinizing hormone signal, to induce hypogonadism and impact sperm quality (see review [14]). The presence of endocrine disruptors such as phthalates in the environment has also been associated with a decrease in sperm quality [15] by the possible antagonist action of phthalates with androgen receptors and enzymes, disrupting steroidogenesis. Steroidogenesis pathways are subject to environmental, pathological, and physiological influences. The monitoring of the extended steroid profile would provide a broader insight into the actual content of steroids in the semen. Hence, this application aims to demonstrate how DynaStI provides useful annotations when analyzing biological samples, as required for an eventual analysis leading to a better understanding of the underlying metabolic pathways.

### 4.1. Materials and Methods

#### 4.1.1. Reagents

Chemicals such as formic acid (FA) were purchased from Biosolve (Dieuze, France); acetonitrile (MeCN), water ($H_{2}O$), and methanol (MeOH) from Fisher Scientific (Loughborough, UK); and phosphoric acid ($H_{3}{\mathrm{PO}}_{4}$) and ammonium hydroxide ~25% from Sigma Aldrich (Wesel, Germany). Steroid standards were obtained either from Sigma Aldrich (Wesel, Germany) or Steraloids (Newport, RI, USA).

#### 4.1.2. Preparation of Samples and Standards

Stock solutions of steroid standards were prepared at 1 mg/mL in methanol. A mix of several steroids was prepared and diluted in $H_{2}O+\mathrm{MeOH}\;\left(95:5\right)$ with 0.1% FA. Working concentrations ranged from 0.1 μg/mL up to 0.2 μg/mL according to the ESI response of each steroid when analyzed independently.

Semen samples were retrieved from healthy donors. After 45 min of liquefaction, the samples were centrifuged 10 min at 3000 rpm to separate seminal liquid from the spermatozoa. Each sample followed an SPE protocol using HLB PRiME µElution plates (Waters, Milford, MA, USA). Briefly, 200 μL of seminal liquid was mixed with 550 μL of $H_{2}O$ containing $4\%\;H_{3}{\mathrm{PO}}_{4}$ on a Thermomixer Comfort (Vaudaux-Eppendorf AG, Schönenbuch, Switzerland) at 450 rpm for 15 min. The samples were then loaded onto the plate and put under positive pressure using a PRESSURE+ 96 manifold (Biotage, Uppsala, Sweden). Then, 400 μL of $H_{2}O/\mathrm{MeOH}\left(95:5\right)+0.1\%\;{\mathrm{NH}}_{4}\mathrm{OH}$ washing solution were passed through each well. Finally, the loaded samples were eluted with 50 μL of MeCN/MeOH (9:1) with a fast step of positive pressure and evaporated using a Speedvac concentrator without heating (LTSC210ARC, Thermo Fischer Scientific, Waltham, MA, USA). Evaporated samples were stored at −80 °C and reconstituted with $100\;\mu L\;\mathrm{MeOH}/H_{2}O\;\left(1:1\right)$ prior to injection into the LC–MS.

#### 4.1.3. LC–MS Workflow

In brief, 10 μL of either samples or a mix of standards were injected into an Acquity H-Class UPLC System (Waters, Milford, USA) equipped with a Kinetex $C_{18}$ column (2.1×150×1.7 mm, from Phenomenex, Torrance, CA) and a pre-filter and maintained at 30 °C. The chromatography gradient is 25 min long and goes from 2% to 100% of mobile phase B over 14 min, followed by a *plateau* of 100% B for 3 min and a reconditioning step of 2% B for 8 min. Mobile phase A was $H_{2}O$ and mobile phase B was MeCN, both containing 0.1% formic acid. The dead time was 0.8 min, and the dwell volume was 0.375 mL. This volume was determined by monitoring with the photodiode array detector the absorption of acetone added to water in a $H_{2}O/\mathrm{MeCN}$ gradient. The dwell time is derived from the middle points of the inflexions in the curve, giving the dwell volume as the product of the dwell time by the flow rate. These conditions include, of course, the previously indicated conditions under which DynaStI accepts its input. The gradient is different from the ones used in the original determination of the experimental $\left(\log k_{w},S\right)$ values for DynaStI [7] so that we can provide an independent example.

Mass spectrometry analyses were performed on a Q-Exactive Focus (Thermo Fischer, Waltham, USA) system equipped with an ESI source (HESI II probe) running in positive mode. The detection of steroids was achieved in full-scan mode with 35,000 resolution at 200 *m/z*. The probe heater temperature was set at 425 °C, and the capillary temperature was set at 300 °C. Auxiliary gas, sheath gas and sweep gas were set to 30, 30, and 3 A.U. The ion spray voltage was 3 kV, and the S-lens $R_{f}$ level was set to 50%. An acquisition range from 120 to 1000 *m/z* was used with an acquisition target (AGC) of $3\times 10^{6}$ ions and a maximum filling time of the C-Trap (IT Fill Time) of 250 ms.

#### 4.1.4. Pre-Processing of Data

Raw files were imported into Progenesis QI (Version 2.3.6275, 64-bit, Nonlinear Dynamics, Newcastle upon Tyne, UK) for automated run alignment, peak picking, and sample normalization. Five adducts were chosen for the peak-picking step and feature grouping $\left({\left[M+H\right]}^{+},{\left[M-H_{2}O+H\right]}^{+},{\left[M-2H_{2}O+H\right]}^{+},{\left[M-3H_{2}O+H\right]}^{+},{\left[M+Na\right]}^{+}\right)$. The list of features (including *m/z*, retention time, adduct information and chemical formula retrieved from the isotopic pattern) were exported as a CSV file and then uploaded to DynaStI for annotation.

### 4.2. Prediction Error

**Figure 1:**
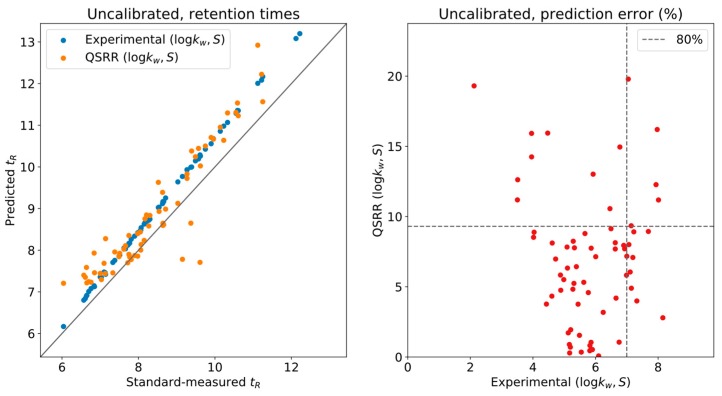
Uncalibrated retention times.

To assess the actual reliability of the predictions, we compared the measured retention times of the standards in the original conditions with those derived from the LSS model for a set of 70 compounds (see Supplementary material) corresponding to the ones for which LSS parameters were available from both experimental and QSRR sources [7]. In Figure 1, we plot the measured/predicted retention time comparison on the left and the relative error of the predictions (w.r.t. the actual measurements) on the right, when done without the calibration. For every compound in the standards, two points are given in the left plot, one with the experimental prediction and one with the QSRR prediction. The relative errors of both are used as the coordinates in the right plot. Ideally, the retention times should fall exactly on the diagonal line, which would correspond to a perfect prediction. As indicated by the obtained slope, a systematic error was present. As previously discussed, this error is related to the determination of the parameters of the instrument’s geometry. Recall that the actual experimental system does not have 0-dimensional injection/mixing points, as the usual LSS model considers. For instance, the dwell volume is affected by capillary connections and valves and contributes a significant volume to the system.

**Figure 2:**
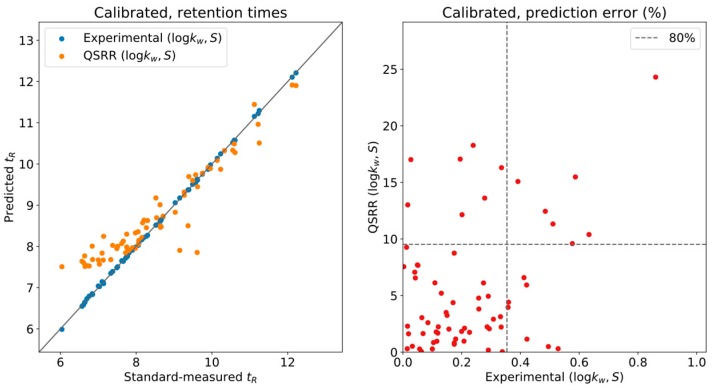
Calibrated retention times.

To correct for this issue, five compounds were used as calibrants for the complete database, namely, in DynaStI’s convention: androstenedione, cortisol, cortisone, testosterone and 4,5α-dihydrotestosterone. It should be noted that the experimentally predicted and QSRR-predicted times have been independently calibrated. As presented in Figure 2, thanks to this automatic internal calibration, and as long as the $\left(\log k_{w},S\right)$ values have been experimentally measured, the LSS model provides exceptional accuracy, with 80% of the values having less than 0.4% deviation between measurement and prediction and none exceeding 1%. QSRR-predicted times show much larger deviations, up to 15%. However, the whole list of QSRR predictions has been calibrated using the QSRR predictions for the five selected compounds. Critically, the error in the predictions used for calibration is carried onto the calibrations themselves, and a systematic deviation is still observed at low retention times.

**Figure 3:**
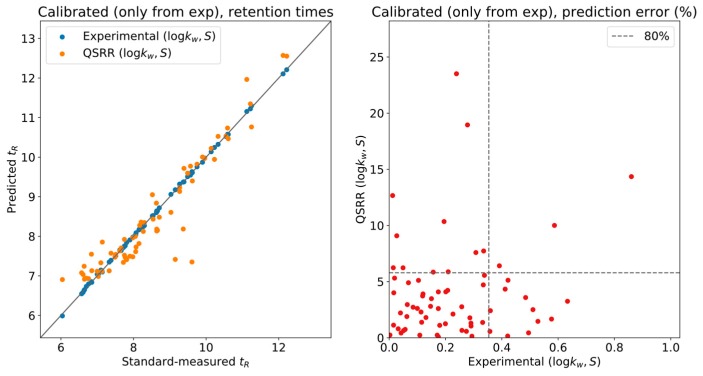
Experimentally calibrated retention times.

To gauge the effect of QSRR error on the calibration, Figure 3 shows experimental and QSRR predictions, both calibrated using only the experimentally determined LSS parameters. Nearly all the systematic deviation in the QSRR predictions of Figure 2 was corrected, and most of the compounds have less than 5% relative error w.r.t. the actual measurement. This is a reasonable tolerance for level two annotations, although it will provide unique results in only a limited number of situations.

In actual use of the DynaStI platform, whenever there are experimentally determined $\left(\log k_{w},S\right)$ values available, they are used instead of QSRR parameter predictions. Concretely, 92 compounds are listed with experimental values, and 106 are listed with QSRR. The whole 198 metabolite database should be calibrated using any selection of compounds with experimental values of $\left(\log k_{w},S\right)$, to avoid propagating the QSRR error to the ensemble of predictions.

### 4.3. Annotation Reliability: Standards

**Figure 4:**
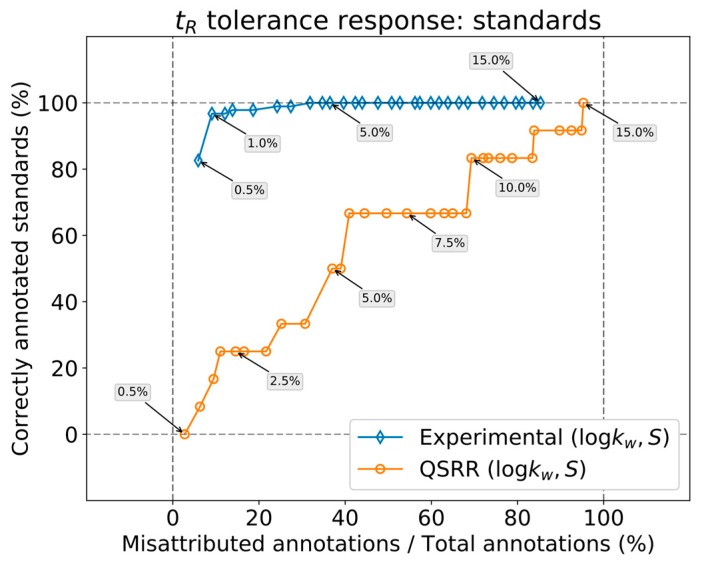
Annotation ratios.

In our previous work, based on preliminary experiments and the literature about the quality of the predictions [7], it was suggested that the tolerance settings for retention times should be 2% and 4%, for experimental and QSRR parameters, respectively. With our analysis, we can go further and optimize these recommended values based on experimental results. Briefly, the findings in the previous section demonstrate that retention time tolerances should be adjusted at 0.5% and 5% for retention times predicted from experimental and QSRR parameters, respectively. These tolerances were established by regarding how many correct annotations were achieved as a function of the tolerance. To establish a quantitative understanding of the effect of tolerance, we have taken the feature list from the measurement of standard analytes and fed it to DynaStI. Of the available analytes in our mix (see Supplementary material), 92 are listed in the DynaStI database with experimental parameters, and 12 are listed with QSRR. The calibrators and the setup are the same as those in the previous section. Because we know exactly the identity of each feature by using standards, we can compute the rate of successful annotations as

(17) $$\frac{features\;annotated\;with\;their\;actual\;identity}{total\;standards\;available\;in\;DynaSti\;database}$$

for each of the two types of prediction. Of course, by increasing tolerance, we also expect to annotate incorrect identities more often. This can be measured by

(18) $$\frac{misattributed\;annotations}{total\;annotations},$$

again for each of the two types. Figure 4 shows these two ratios plotted against each other for tolerances ranging from 0.5% to 15.0%, in a similar fashion to receiver operating characteristic (ROC) plots (notice that annotation is not a binary classification). The immediate takeaway is that there is no reason to use more than 5% tolerance for experimental LSS predictions, and in fact, 1% should suffice in most situations—nearly all of the compounds available in the database are found, without generating too many false annotations. The limit is what we expected from Figure 3, but it is obviously subject to good reproducibility on the measurement side. As previously explained in Section 2, level 2+ annotations at the optimal tolerance level will produce few ambiguities in annotation.

QSRR predictions are a different matter. The evolution of misattributed annotations is more or less similar to experimental predictions since it depends only on how many features the annotation ‘grabs’ for a given tolerance, which in turn is a function of feature density. However, due to the larger error from the QSRR parameters, one should use a tolerance from 10% to 15% to cover most of the compounds in the database. These relatively high tolerance values inevitably produce a high number of misattributed annotations.

### 4.4. Annotation Multiplicity: Real Sample

While working with standards, the list of features is relatively limited, but this is not the case for real untargeted analysis. In the case of the seminal liquid sample application, the peak-picking software was able to determine 21,474 different features from one single LC–MS analysis. Increasing feature density necessarily increases annotation multiplicity for the same tolerance level, which can be decidedly problematic for QSRR predictions if we are to use the ample tolerances suggested in the previous subsection.

Because in a real sample, the real identities of the annotated features remain unknown (which is the whole reason for annotation prior to identification), the ratio of correctly annotated compounds cannot be considered. In any case, once the correct identity is assigned, increasing the tolerance will not remove the corresponding correct annotation. However, given the high number of detected peaks in a real biological sample, the risk of annotating features that are either unknown or noise will directly inflate the rate of misattributions. These issues can manifest in two ways as tolerance is incremented. One is by finding a match for a feature with no previous annotations. Another, more problematic, is by assigning several putative identities to one single feature. The reason the former is preferred is that ‘interesting’ features are commonly selected by other means in a biological study. Finally, other properties could be used to help identity confirmation, such as the matching of MS/MS spectra with a reference database.

**Figure 5:**
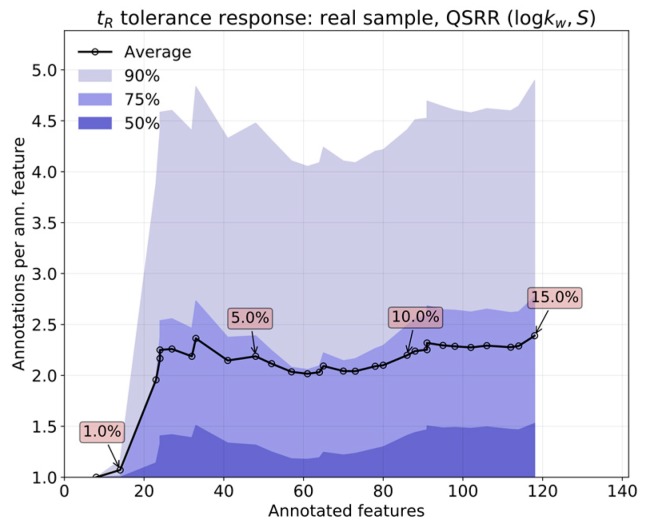
Annotation multiplicity (QSRR).

To quantify the annotation multiplicity in a real sample, we plot in Figure 5 the number of QSRR annotations per annotated feature for a range of tolerances, versus the number of annotated features themselves. The main line represents the average over all the features, and the shades represent the 50%, 75%, and 90% interpolated percentiles. The percentiles have been estimated with a gamma distribution, as it naturally includes many specific cases of non-negative variable distributions, while still being analytically tractable. At the minimal tolerance of 0.5%, compounds were annotated with a unique identity. The number of proposals grows quickly as one opens up to ~2%, with the worst cases presenting up to four putative identities per feature. After ~2%, the number of proposals plateaus, and further increases in tolerance mostly add different features to the set of annotations. Notice that approximately half of the features presented only one annotation. This means that we are in the good scenario of potential wrong annotation, as described before. Even if many of these annotations may not include the real identity of the annotated feature, the relatively low number of proposals could be easily confirmed by Supplementary information (e.g., MS/MS).

**Figure 6:**
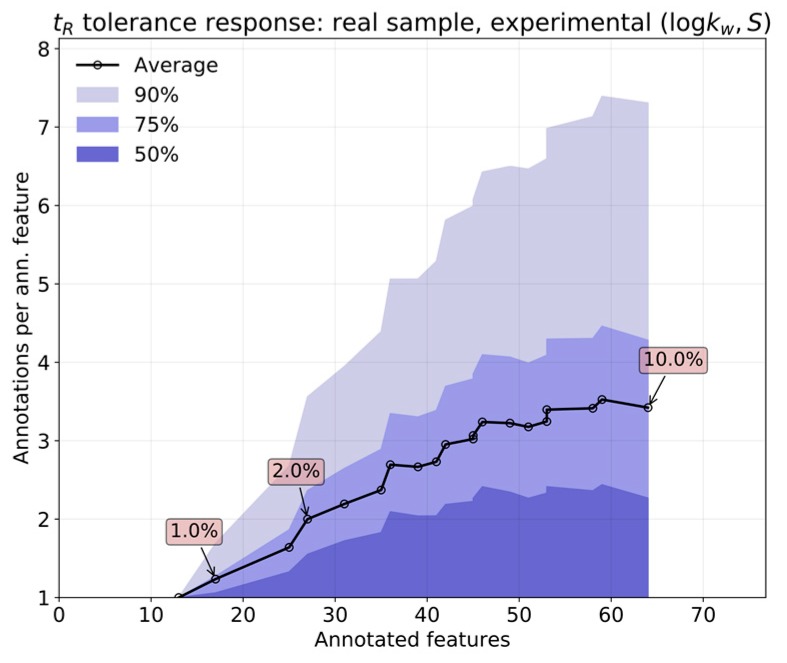
Annotation multiplicity (Exp).

A similar plot is shown in Figure 6 for LSS experimental predictions. In this case, the expected number of annotations per feature is higher than that for QSRR. The library of standards used for the original determination of experimental $\left(\log k_{w},S\right)$ values contains more isomers than those that are present in the QSRR calculations. Consequently, a maximum value of 2% tolerance should be used, even in real samples.

## 5. Conclusions

We have introduced and documented DynaStI, a dynamic retention time database and annotation interface for LC–MS datasets in untargeted steroidomics. DynaStI uses the well-known LSS model to generate retention time predictions for any specific chromatographic gradient and then compares them to a list of experimental features, returning a list of putative identities for each. The parameters of the LSS model have been determined experimentally for a wide range of steroids from standards, and predicted from state-of-the-art *in silico* models for others.

The autocalibration capabilities of the software were evaluated. It can be used to compensate for minor perturbations to the LSS model that arise naturally from inaccuracies in the determination of instrumental parameters. We have observed that the calibration is mandatory to obtain high-quality predictions for nearly-unambiguous annotation.

The difference in prediction power between experimental and *in silico* parameters has been quantified, finding for a set of standards that, once calibrated, deviations between prediction and actual retention time are lower than 1% for experimental parameters and typically lower than 10% for *in silico* parameters. These parameters require the user to employ different retention time tolerances for annotation of each mode of prediction.

To determine these tolerances, we studied the success rate and multiplicity of annotations on standards and on a real biological sample containing steroids (i.e., seminal fluid analysis). We have found that around the levels suggested by the errors in prediction, most or all of the compounds in the database can indeed be annotated by DynaStI. No excessive multiplicity of annotations per feature was generated as a consequence of these tolerance values. This outcome is of importance to avoid too many candidates to test on an eventual identification step.

In summary, we have validated the ability of DynaStI to serve as an annotation database for measurements in an arbitrary LC gradient, allowing the fine-tuning of the separation without the need to measure whole libraries of standards on each iteration. An open issue for the future is the generalization of the method to all metabolites that can be retained by lipophilic interaction and analyzed by RPLC, with a special emphasis on other classes of lipids where the number of isomers is important. Another direction would be to extend the approach and to evaluate the differences observed in similar stationary phase chemistries (i.e., C18), based on the use of a calibration ‘test set’ allowing the rapid obtention of new parameters for the LSS models.

## Supplementary Materials

The following are available online at https://www.mdpi.com/2218-1989/9/5/85/s1. Figure S1: Uncalibrated retention times; Figure S2: Calibrated retention times; Figure S3: Experimentally calibrated retention times; Figure S4: Annotation ratios; Figure S5: Annotation multiplicity (QSRR); Figure S6: Annotation multiplicity (Exp); Table S1. compounds-annotation-standards; Table S2. compounds-prediction-error.
