# Supplementary material for: DynaStI: A Dynamic Retention Time Database for Steroidomics
Source: Metabolites. 2019 Apr 30;9(5):85. doi: 10.3390/metabo9050085 (PMC6572260; doi:10.3390/metabo9050085)
Supplement: Supplementary file 1 [file metabolites-09-00085-s001.zip › dynasti-detailed-figure-captions.pdf]

DynaStI – Detailed figure captions

Figure 1. Uncalibrated retention times.

The left plot shows for each compound the retention time predicted by the LSS model from the parameters in the database vs. measured retention times. The straight line represents a perfect prediction. The right plot shows the relative prediction error (the relative distance to the straight line on the left plot) for both the LSS values predicted from experimental LSS parameters and for QSRR LSS parameters.

Figure 2. Calibrated retention times.

Same as Figure 1, but with the predictions corrected by the calibration algorithm described in section 3.2. We can observe that the predictions lie closer to the straight line representing a perfect prediction, although there is a noticeable non-linearity in the predictions from QSRR LSS parameters.

Figure 3. Experimentally calibrated retention times.

Same as Figure 2, but both the predictions from experimental LSS parameters and from QSRR LSS parameters have been calibrated using for the calibrators predictions from experimental LSS parameters. In particular, the errors in the right plot now gather around zero for both kinds of predictions. This highlights the importance of using compounds with available experimental LSS parameters for the calibration.

Figure 4. Annotation ratios.

For increasing levels of annotation tolerance using both predictions from QSRR and experimental LSS parameters, marked as labels in the plot, we show the fraction of correctly annotated compounds over the total of compounds (i.e. whose actual identity has been found amongst the annotations) vs the fraction of misattributed annotations over the total (i.e. annotations that do not correspond to the actual identity of their assigned compound). Higher tolerances naturally increase the number of misattributions, and the main feature of the plot is that predictions from experimental LSS parameters achieve very fast a high rate of correct annotation. Again, this highlights the robustness of the LSS model when provided with experimental parameters.

Figure 5. Annotation multiplicity (QSRR).

For increasing levels of annotation tolerance using predictions from QSRR LSS parameters, marked as labels in the plot, we show the average and distribution (by coloring certain quantiles) of average number of annotations per feature, versus the absolute number of features that get an annotation. Notice that the average can go down if increasing the tolerance makes the algorithm take in feature that previously had no annotations.

Figure 6. Annotation multiplicity (Exp).

Same as Figure 6, but using predictions from experimental LSS parameters.
