# Supplementary material for: DynaStI: A Dynamic Retention Time Database for Steroidomics
Source: Metabolites. 2019 Apr 30;9(5):85. doi: 10.3390/metabo9050085 (PMC6572260; doi:10.3390/metabo9050085)
Supplement: Supplementary file 1 [file metabolites-09-00085-s001.zip › dynmetid-command-line-reference.pdf]

# DynaStI – Command line parameters for the DynMetID core

DynMetID can be run as a command line command line tool with the following parameters

```
>dynmetid <database host>...
     <database user> <password>...
     <database name> <compound property table>...
     <feature list file> <adduct list file>...
     <mass tolerance ppm>...
     <exp. ret. time tol. %> <QSRR ret. time tol. %>...
     <gradient start %> <gradient end %>...
     <gradient time min> <flow rate mL/min>...
     <dead time min> <dwell volume mL> [calibration list file]
```

The database must be an SQL database, with a table of compounds with the fields `name`, `logkw` and `s`, corresponding to each of the relevant parameters of the compound. The concrete database measured and curated in the context of the accompanying article is hosted at `dynasti.vital-it.ch`, as part of the Vital-IT infrastructure. The feature list must be a path to a plain-text file containing single line entries of the form

```
<retention time min>_<m/z of ion>m/z
```

or

```
<retention time min>_<exact neutral mass>n
```

in the case of features for which the neutral mass is already known. The adduct list must equally point to a plain-text file with entries providing

```
<adduct name>;<adduct mass excess>;<adduct charge>
```

Finally, the optional calibration list file should contain entries with

```
<compound name>;<experimental retention time min>
```

The name of the compound must, of course, match one of those in the database.
